# Application and Characterization of Gum from *Bombax buonopozense* Calyxesas an Excipient in Tablet Formulation

**DOI:** 10.3390/pharmaceutics4030354

**Published:** 2012-08-03

**Authors:** Ndidi C. Ngwuluka, Jehu Kyari, John Taplong, Onyinye J. Uwaezuoke

**Affiliations:** 1 Department of Pharmaceutics and Pharmaceutical Technology, Faculty of Pharmaceutical Sciences, University of Jos, Jos, 930001, Nigeria; Email: jevelyk@yahoo.com (J.K.); johntapsy@yahoo.com (J.T.); 2 Department of Pharmaceutics and Pharmaceutical Technology, Faculty of Pharmacy, Olabisi Onabanjo University, Ago-Iwoye, 120005, Nigeria; Email: onyinyemeka@gmail.com

**Keywords:** tablets, tablet manufacture, natural gums, *Bombax buonopozense*, drug delivery, zero order, controlled release, dosage forms, oral delivery

## Abstract

This study was undertaken to explore gum from *Bombax buonopozense* calyxes as a binding agent in formulation of immediate release dosage forms using wet granulation method. The granules were characterized to assess the flow and compression properties and when compressed, non-compendial and compendial tests were undertaken to assess the tablet properties for tablets prepared with bombax gum in comparison with those prepared with tragacanth and acacia gums. Granules prepared with bombax exhibited good flow and compressible properties with angle of repose 28.60°, Carr’s compressibility of 21.30% and Hausner’s quotient of 1.27. The tablets were hard, but did not disintegrate after one hour. Furthermore, only 52.5% of paracetamol was released after one hour. The drug release profile followed zero order kinetics. Tablets prepared with bombax gum have the potential to deliver drugs in a controlled manner over a prolonged period at a constant rate.

## 1. Introduction

Excipients are the non-therapeutic but vital components of drug delivery systems. They influence drug delivery through increased/decreased solubility, modified dissolution rates, absorption enhancement, ultimately leading to improved therapeutic activity and even a decrease of unwanted side effects [1].

The cost of drug development drives the quest to search for low-cost ingredients and enabling companies to enhance their existing products as well as to develop new drug delivery systems in order to cope with the global challenges and competition. Novel excipients enable pharmaceutical companies to develop new drug delivery systems, improve efficiency, enhance functionality and reduce the cost of drugs [2]. Furthermore, with more drug patents set to expire in the next three to four years, novel excipients offer patent holders opportunities to upgrade their products and thereby extend their patent lives. 

Development of excipients from natural sources which are known to be utilized for food consumption may reduce the regulatory requirements for approval. Excipients from plant sources would be cost and environmentally friendly due to the availability of plants, low or no toxicity and biodegradability. Even agricultural wastes such as corn stalk, rice hulls and orange mesocarp have been recycled and microcrystalline cellulose produced from them [3]. Excipients from plant sources are appealing because plant resources are renewable and if maintained and harvested in a sustainable manner, they can be constant sources of raw materials [4].

*Bombax buonopozense* from family Bombacaceae is a wild tree found in Nigeria mainly in the North-Central part of Nigeria. The indigenes eat the floral part of the plant and it is also known for its medicinal use. It is also found in other West African countries such as Ghana [5,6], Gambia [7] and Côte d’Ivoire [8]. The dried stem is used in the treatment of malaria in Ghana by boiling in water and drinking [9]. The bark is used to treat chest pain in Gambia [7]. The decoction of the leaves is used to manage stomach ulcers and burns in Ghana [10]. 

Various extracts of the floral parts were investigated for antimicrobial activity and were found to be active against the organisms (*Staphylococcus aureus*, *Escherichia coli* and *Aspergillus niger*) investigated [11]. The seed has also been documented for its antimicrobial activity [12]. The red calyxes which are mucilaginous and the immature fruits are used in cooking soup or sauce [13]. In Nigeria, different parts of *Bombax buonopozense* are employed for various purposes. The immature fruits are prepared as an emollient; decoction of young leaves is used as a warm bath for febrile children; the ground bark is taken by pregnant women to increase lactation; the extract from the bark is drunk or applied on the head for dizziness; and the gum resin from the bark is pulverized, mixed with oil and used to manage skin diseases such as “craw-craw”. However, little or no information is known concerning use of gum from *Bombax buonopozense* dried calyxes as a pharmaceutical excipient.

Therefore, this study explored the use of gum from *Bombax buonopozense* calyxes as a binding agent using the wet granulation method. The techniques used to characterize the tablets were the compendial and non-compendial tests such as hardness test, uniformity of weights, disintegration and *in vitro* drug release studies.

## 2. Results and Discussion

**Figure 1:**
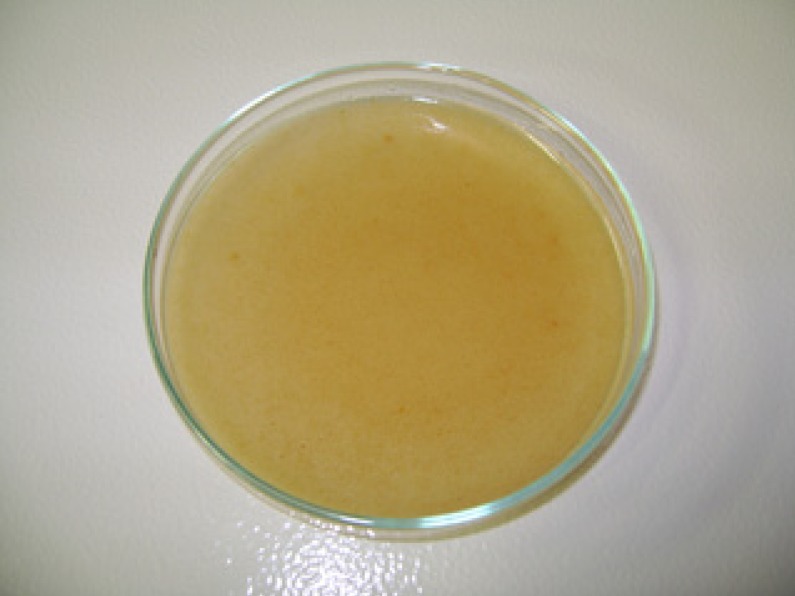
Digital image of bombax gum dispersed in water.

The gum (bombax gum) obtained was brown in color and slightly gritty to touch. The percentage yield obtained after extraction from the calyxes was 8.02%. The phytochemical analysis indicated the presence of protein, starch and tannins. The gum when dispersed in water is as shown in Figure 1.

### 2.1. Viscosity Analysis of Gum from Dried Calyx of *Bombax buonopozense*

**Figure 2:**
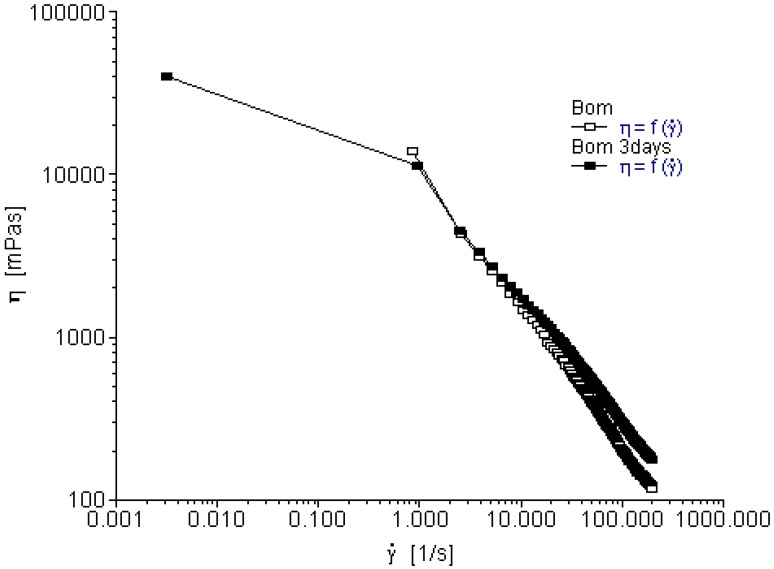
Viscosity as a function of shear rate for bombax gum.

Bombax gum exhibited non-newtionian pattern of flow; as the shear rate increased, the viscosity decreased. It also exhibited shear thinning as shown in Figure 2. The alignment of the profiles from the first and fourth days indicates the bombax was basically stable after three days of storage. After three days of storage, bombax gum neither hydrolyzed nor was its viscosity affected by ambient temperature used during storage. 

### 2.2. Characterization of Granules

**Figure 3:**
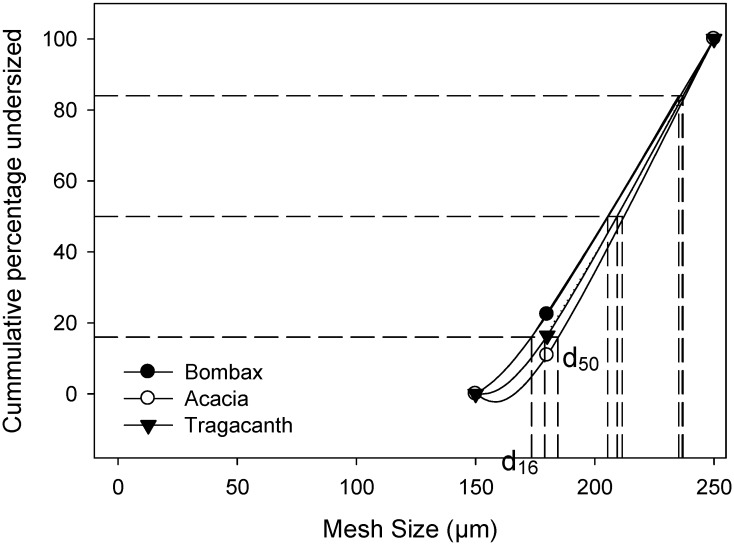
Particle size distribution of granules prepared from different binders.

Granules are characterized to ensure their suitability for tableting in order to produce tablets with content uniformity, uniformity of weights and are able to withstand pressure. Particle size distribution of granules influences the performance of tablets as it impacts on flowability, content and weight uniformity; compactibility and rearrangement of particles as well as drug release [14,15]. The curve in Figure 3 shows cumulative percentage undersize which was obtained by plotting cumulative weight percent against mesh size. The curve also indicated the undersize and oversize, 16% and 84% of the particles. The granules prepared with different binders had particles with sizes greater than 150 µm while over 60% of the particles sizes were greater than 180 µm. Consequently, the mean weight particle size for bombax, acacia and tragacanth were 205.40 µm, 211.54 µm and 209.87 µm respectively. It was apparent that the granules were more coarse than fine. However, fine particles are indicative that the tablets produced may be hard and less friable as there will be less unfilled voids during the compaction process. 

The angle of repose, Carr’s compressibility and Hausner’s quotient explicate the compressibility of the granules to produce acceptable tablets. All batches with different binders exhibited good flow rate (Table 1). Granules with acacia gum as binding agent had a higher flow rate while those of bombax gum had the least. The flow rates of the granules prepared with the different binders were statistically significant (*p* = 0.024), at a significance level of 0.05. The angle of repose for granules with bombax gum was less than 30° which confirms that the granules had good flow properties. Generally, granules with an angle of repose less than 30° [16], Hausner’s quotient below 1.25 [17], and Carr’s compressibility below 25% [18] exhibit good flow properties. Granules prepared with bombax gum exhibited good flow properties, better than those of granules prepared with either acacia or tragacanth gum, as shown in Table 1. The difference in angles of repose obtained for the granules prepared with different binders were significant (*p* = 0.039) while the differences between the binders for densities, Hausner’s ratios and Carr’s compressibilities were not significant. The good flow properties of granules with bombax gum indicate that there may be insignificant or no weight variability [19] as the granules will take less time to fill the die cavities. 

**Table 1:** Analysis of granules prepared with different binders.

Test		Binder	
	Bombax gum	Acacia gum	Tragacanth gum
Flow rate (g/sec)	6.15 ± 0.05	9.80 ± 0.10	7.75 ± 0.05
Angle of repose (°)	28.60 ± 0.10	26.50 ± 0.15	25.20 ± 0.10
Bulk density (g/mL)	0.487 ± 0.01	0.476 ± 0.01	0.465 ± 0.01
Tapped density (g/mL)	0.6200 ± 0.01	0.6250 ± 0.005	0.6061 ± 0.01
% compressibility	21.30 ± 0.35	23.80 ± 0.36	23.30 ± 0.4
Hausner’s quotient	1.27 ± 0.03	1.31 ± 0.03	1.30 ± 0.03

### 2.3. Evaluation of Tablets Properties

Uniformity of weights, thickness and diameter are indications of the uniformity of the active ingredient in a tablet batch. The uniformity of weights compendial specification states that for tablets weighing more than 324 mg, weights of not more than two tablets should deviate from the average weight by more than 5% [20]. The tablets of the different binders met this specification. 

Friability and hardness tests are compendial and non-compendial mechanical properties used to assess the ability of tablets to withstand pressures or stress during handling, packaging and transportation. Hardness of a tablet is known to influence the rates of disintegration and dissolution. The minimum required tablet hardness is 4 kp [21] and tablets formulated with the different binders met the minimum requirement and those of bombax gum were hardest, as shown in Table 2. While hardness test relates to bulk deformation of tablets, friability relates to the surface characteristics which in turn influence dissolution [22]. The acceptable specification for friability of tablets is not more than 1% [20]. The friability of the tablets was above the specification; however, this may be improved by increasing the concentration of the binders. Tragacanth is a soft binder and so was more friable than acacia and bombax gums. 

**Table 2:** Evaluation of tablets based on compendial and non-compendial tests.

Test		Binder	
	Bombax gum	Acacia gum	Tragacanth gum
Friability (%)	3.10 ± 0.03	7.54 ± 0.03	9.98 ± 0.03
Mean Hardness (kp) ^1^	6.3 ± 0.27	5.5 ± 0.20	5.3 ± 0.1
Disintegration time (Minutes)	>60.00	4.00 ± 0.17	2.00 ± 0.1
Uniformity of weight	660 ± 0.21	660 ± 1.1	634 ± 1.8
Mean Diameter (mm)	11.20 ± 0.1	11.20 ± 0.1	11.20 ± 0.1
Mean Thickness (mm)	3.5 ± 0.05	3.5 ± 0.1	3.5 ± 0.06
% drug release at T_45_	33.90 ± 0.63	88.14 ± 0.60	97.5 ± 0.61
% drug release at T_60_	52.50 ± 0.7	94.0 ± 0.75	100.8 ± 0.8

^1^ kp iskiloponds.

The tablets formulated with acacia and tragacanth gums disintegrated in less than five minutes while those with bombax gum did not disintegrate after one hour. Hence tablets with bombax gum did not comply with the compendia specification for disintegration of uncoated immediate release dosage forms. The mechanism of disintegration by maize starch, the disintegrant incorporated into the formulation (Section 3.4, Table 5), is based on penetration of water into the tablet; swelling of disintegrant with development of swelling force resulting in a repulsive force between the particles. Disintegration can also be enhanced if the binders are self-disintegrating and the penetrating solvent has a high dielectric constant that can weaken the intermolecular bonds within the particles. Hence, the disintegration of tablets with bombax gum may have been prevented by the binder cohesive forces being greater than the disaggregating forces produced by maize starch swelling upon hydration. Furthermore, hydration of the tablets led to gel formation on the outer core of the tablets and gel formation may have prevented hydration of the inner core and the anticipated disintegration of tablets. The non-disintegrating behavior of tablets formulated with bombax gum suggests bombax gum’s possible potential as a sustained release drug carrier. Statistically, the friability, disintegration and uniformity of weight of the tablets with the different binders were significantly different (*p* = 0.034; *p* < 0.05 and *p* < 0.05 respectively) while the hardness, uniformity of diameter and thickness were not significantly different at a significance level of 0.05. 

**Figure 4:**
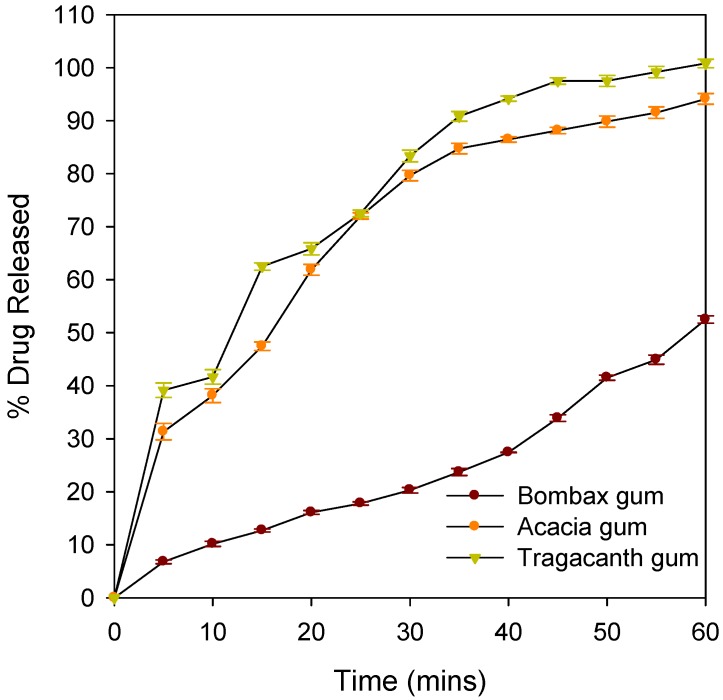
Drug release profiles of tablets formulated with bombax gum, acacia gum and tragacanth gum.

*In vitro* drug release studies portrayed that bombax gum may have a potential for controlled released delivery of drugs. Only about half of paracetamol was released after one hour. At T_45,_ tablets with acacia gum had released over 85% and tragacanth had released over 95% of paracetamol while bombax released about 34%. The percent dissolved showed significant statistical difference (*p* < 0.05) between the tablets of different binders. This study implies that the concentration employed for binders (3.5%) which were suitable for acacia and tragacanth gums could not be used for bombax gum to formulate an immediate release dosage form. A lower concentration of bombax with endo- and exogenous disintegrants may be explored for immediate release dosage forms. However, a lower concentration may not yield hard and less friable tablets. Figure 4 shows the drug release profiles of tablets with the different binders over 60 min indicating the influence of the binders on the rate of drug release. Mathematical models such as dissolution efficiency (DE) (Equations 1 and 2), zero and first orders were used to compare the release profiles (Table 3).
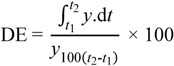
(1)where *y* is the percentage dissolved at time *t.*

The integral of the numerator which is the area under the curve was calculated using the trapezoidal method:

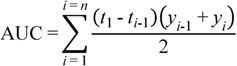
(2)

**Table 3:** Outcomes of mathematical modeling of drug release profiles.

Binder	Zero Order			First Order			DE (%)
	*r*	*r* ^2^	*K*_0_	*r*	*r* ^2^	*K*_1_	
Bombax Gum	0.9835	0.9673	0.7947	0.9588	0.9192	−0.0049	44.65
Acacia Gum	0.9228	0.8516	1.3804	0.9923	0.9846	−0.0196	72.48
Tragacanth Gum	0.9184	0.8435	1.4375	0.9803	0.9609	−0.0367	73.95

The difference in dissolution efficiency of tablets prepared with acacia and tragacanth gum is about 1.47% indicating that these binders can be used interchangeably in the formulation of paracetamol tablets (a difference between dissolution efficiencies of less than 10% is assumed to indicate bioequivalence, [23]). On the other hand, bombax gum had a less desirable dissolution efficiency of 44.65%, suggesting that, while this binder cannot be used interchangeably with acacia or tragacanth gums (difference larger that 10%) in immediate release formulations, it may have a potential use in the manufacturing of sustained release formulations. The kinetic model that best fits the drug release profiles of tablets of acacia and tragacanth gums was the first order kinetics while that of bombax gum was zero order kinetics. This is an indication that bombax gum may be employed to produce tablet matrices that can deliver drugs at a fairly constant rate over a prolonged period. 

Furthermore, Higuchi and Korsmeyer-Peppas equations were employed to analyze the drug release data. Higuchi equation which is based on Fick’s law of diffusion is:

*Q* = *K*_H_*t*^1/2^ (3)

where Q is the amount of drug released from the drug carrier over time and *K*_H_ is the Higuchi dissolution constant which can be obtained by plotting cumulative percentage over square root of time.

Korsmeyer-Peppas equation is used to explain the drug release mechanism from a drug carrier and is expressed as:

log[*Q_t_/Q_∞_*] = log*K* + *n* log*t* (4)

where *Q_t_/Q_∞_* is the amount of drug released per time; *K* is the constant and *n* is the release exponent and these can be obtained by plotting log of percentage drug release over log of time [24]. The outcomes of Higuchi and Korsmeyer-Peppas equations are shown in Table 4.

**Table 4:** Outcomes of mathematical modeling of drug release profiles.

Binder	Higuchi			Korsmeyer-Peppas			*n*
	*r*	*r* ^2^	*K*_H_	*r*	*r* ^2^	*K*_KP_	
Bombax Gum	0.9271	0.8056	4.983	0.9877	0.9727	0.471	1.136
Acacia Gum	0.9858	0.9705	13.154	0.9875	0.9750	15.824	0.449
Tragacanth Gum	0.9866	0.9678	14.275	0.9905	0.9811	19.437	0.415

In addition of Higuchi and Korsmeyer-Peppas equations to zero and first orders, the best fit models for the gums were: Korsmeyer-Peppas equation for bombax gum; first order for acacia gum and Korsmeyer-Peppas equation for tragacanth gum. The release exponent *n* when applied to a cylindrical matrix indicates that the release mechanism is Fickian diffusion if *n* = 0.45; non-Fickian release or anomalous transport if 0.45 < *n* < 0.89; Case II transport or zero order release if *n* = 0.89 and Super Case II transport if greater than 0.89 [24]. Mechanism of release from acacia gum on application of the release exponent *n* could be said to be Fickian diffusion as *n* = 0.449 while that of bombax gum could be said to be Super Case II transport as *n* is greater than 0.89.

## 3. Experimental Section

### 3.1. Materials

Acacia (BDH Chemical Ltd, Poole, England), tragacanth (Steculia Gum, Halewood Chemicals Ltd, Stanwell Moor, Staines, Middlesex, England), Paracetamol BP (China), maize starch (G Koepcek E & Co GMBH), talc (Hopkins and Williams, Chadwell Health Essex, England), Magnesium stearate (Gurr Chemicals, Bell Sons & Co, Southport, England). All other reagents were of analytical grade and used as received.

### 3.2. Extraction of Gum from Dried Calyx of *Bombax buonopozense*

The dried calyxes of *Bombax buonopozense* were grinded and 350 g was transferred into a bowl. Five liters of distilled water were added into the bowl and stirred continuously until mucilage was formed. The mucilage was separated from the calyxes with a fine muslin bag and the gum was precipitated by washing the mucilage several times under agitation with absolute alcohol. Afterwards, the gum was air-dried for 24 h, weighed and milled. The percentage yield was thereafter calculated.

### 3.3. Viscosity Determination of Gum from Dried Calyx of *Bombax buonopozense*

Haake MARS rheometer (Thermo Electron Corporation, Kalrushe, Germany) in oscillation mode, fitted with a cone and plate geometry with a diameter of 35 mm and cone angle of 1°—sensor C35/1°Ti was employed and the data was obtained using RheoWin PC 3 software. Viscosity was determined by subjecting bombax gum to shear rate range from 0.00–200.00 s^−1^ over 180 s. After the first day of viscosity testing, the gum was stored and the test was repeated after 3 days (on the 4th day).

### 3.4. Preparation and Characterization of Granules

Paracetamol granules were prepared by wet granulation with maize starch as diluent and disintegrant, talc as glidant and magnesium stearate as lubricant. The composition of the granules batches is shown in Table 5 and the targeted weight of each tablet to be compressed from the granules was 635 mg. Acacia and tragacanth gum were used as binders for comparative studies with bombax gum. The granules were characterized by determining flow rate, angle of repose, bulk and tapped density using the procedures described by Ngwuluka and co-workers [14]. Particle size analysis was undertaken by mesh analysis using a stack of three sieves (sieve No 60, 80 and 100). The granules were weighed, placed on the top sieve (No. 100) and shaken for 10 min. The quantities in each sieve were obtained gravimetrically. 

**Table 5:** Composition of granules for tablet formulation.

Components	Percentage
Maize starch	15%
Binder	3.5%
Talc	2%
Magnesium stearate	0.5%
Paracetamol	79%

### 3.5. Granules Compression and Tablet Evaluation

The granules were compressed using a single punch die set of 13 mm with a fairly uniform compression pressure of 467 Pa. The die cavity was set to obtain the required weight of the tablets. The tablets were evaluated by undertaking both compendial and non-compendial tests in order to assess the quality and performance of the tablets with the different binders and these tests included hardness test, uniformity of weights, friability test, disintegration test, thickness and diameter. The procedures for the tests are as stated by Ngwuluka and co-workers [14]. 

### 3.6. *In vitro* Drug Release Studies

The *in vitro* drug release studies were carried out with USP apparatus II (RC-6 dissolution testing apparatus, Genlab Ltd, England) at stirring rate of 50 rpm and temperature of 37 ± 1 °C. The dissolution medium was 1000 mL 0.1 N HCl and samples were withdrawn at 5 min intervals for one hour. Equal volume of fresh medium was replaced after each withdrawal in order to maintain sink conditions. The concentrations of samples per time interval were determined spectrophotometrically after filtering at 290 nm. 

### 3.7. Data Analysis

Simple statistical analysis such as standard deviation; and analysis of variance (ANOVA) were used to compare the data obtained for the different binders while dissolution efficiency, first and zero orders, Higuchi and Korsmeyer-Peppas equations were used for *in vitro* drug release studies.

## 4. Conclusions

Bombax gum was tested as a binding agent in formulations used to make immediate release paracetamol tablets. Tablets formulated with bombax gum showed acceptable flow properties, better than comparable binders such as acacia and tragacanth gums. However, upon hydration, tablets did not disintegrate as promptly, rather showing a tendency to act as sustained release tablets. This behavior suggests that, if the quantity of bombax is increased and lower dose drugs are used, it may be possible to use this gum as a release sustaining agent, with release kinetics close to zero order. Also, this material could be an effective release modulator to bring the release kinetic of well known, mainly first order release sustaining carriers, such as HPMCs, close to, in most cases, a more desirable zero order. Therefore, bombax gum shows promise to become a widely available, non-toxic, biodegradable, excipient that can be advantageously used for the preparation of effective sustained release tablets. The ability of bombax to perform this way will be further explored and will be the subject of a future paper.
